# Lorentz Force Actuated Tunable-Focus Liquid Lens

**DOI:** 10.3390/mi10100714

**Published:** 2019-10-22

**Authors:** Kari L. Van Grinsven, Alireza Ousati Ashtiani, Hongrui Jiang

**Affiliations:** 1 Department of Electrical and Computer Engineering, University of Wisconsin-Madison, Madison, WI 53706, USA; kvangrinsven@wisc.edu (K.L.V.G.); ousatiashtia@wisc.edu (A.O.A.); 2 Department of Materials Science and Engineering, University of Wisconsin-Madison, Madison, WI 53706, USA; 3 Department of Ophthalmology and Visual Sciences, University of Wisconsin-Madison, Madison, WI 53706, USA; 4 Department of Biomedical Engineering, University of Wisconsin-Madison, Madison, WI 53706, USA; 5 McPherson Eye Research Institute, University of Wisconsin-Madison, Madison, WI 53706, USA

**Keywords:** tunable lens, liquid lens, magnetic actuator, optofluidics

## Abstract

Tunable-focus liquid lenses provide focal length tuning for optical systems, e.g., cameras, where physical movement of rigid lenses are not an option or not preferable. In this work we present a magnetically actuated liquid lens utilizing the Lorentz force to vary the focal length as the current through the system is varied. The resulting lens can operate as both a diverging and a converging lens depending on the direction of current applied and has a large range of focal lengths, from −305 mm to –111 mm and from 272 mm to 146 mm. We also characterized the aberrations of the lens during the actuation with a Shack–Hartmann wavefront sensor, and utilized the lens for imaging, during which we measured a resolution of 7.13 lp/mm.

## 1. Introduction

Tunable lenses are a class of optical lenses that provide adjustable focal lengths, as compared to the fixed focal lengths found in traditional solid lenses. This tunability provides an extra degree of freedom for designers of optical devices. In addition, imaging devices with a tunable lens do not need a translational lens movement to focus, as the focusing can be done by tuning the focal length itself. Not having a moving lens barrel found in most of traditional imaging devices results in a faster response time, a more compact package, and a robust device structure [1]. To realize focus tunability, various mechanisms, for example, liquid crystal [2,3,4], polymer [5,6,7], and most frequently, liquid-filled focus tunable lenses [1], have been proposed and used.

Tunable-focus liquid lenses utilize the different indices of refraction of different liquids in order to form a lens at the interface of these two liquids. Generally, this is done in one of two ways, either by using two immiscible liquids or by employing a membrane. In the case of a membrane, the membrane may be used between liquid and air, or between two different liquids. While liquid lenses without a membrane generally rely upon electromagnetic or thermal properties of the liquids themselves in order to actuate the lens [8,9,10,11,12,13,14], liquid lenses formed with the addition of a simple membrane can be actuated through a much wider range of mechanisms. Thanks to the near-incompressibility of liquids, these lenses can utilize a variety of different actuators, including pneumatic [15,16,17,18,19], electrowetting [20,21,22,23,24], magnetic/electromagnetic [25,26], piezoelectric [27,28,29,30,31,32], electrostatic [33], dielectrophoretic [11,34], or any other actuator that can be coupled to a sealed liquid chamber to provide focus tuning. Electromagnetic actuators are particularly appealing because they can enable extremely fast response times on the order of 2–3 ms [35,36]. Electromagnetic actuators can also apply large enough forces to a system to enable relatively large deformation of the membrane that defines the lens, and these large deformations translate into a larger range of potential focal lengths of the liquid lens.

Using electromagnetism and taking advantage of the inherent relationship between electricity and magnetism in order to achieve linear actuation has been taking place for decades. It is the basis of such well-established technology like the voice coil actuator, which utilizes an electrical coil and a permanent magnet to translate the movable portion of the system into a piston motion [25,26,37]. Electromagnetism has been used as the actuating force for a number of different kinds of tunable lenses and optical systems as well, though there is a large variety of configurations of electromagnet/wiring and permanent magnet [38,39,40]. Some designs use a membrane to transfer the actuation from one region of a chamber to another in order to ensure a clear optical path for the lens. In our design, we have implemented a simple magnetic actuator that relies on radial magnetic field, which reduces the complexity of the magnetic circuit that we will describe next.

## 2. Actuation Mechanism

**Figure 1:**
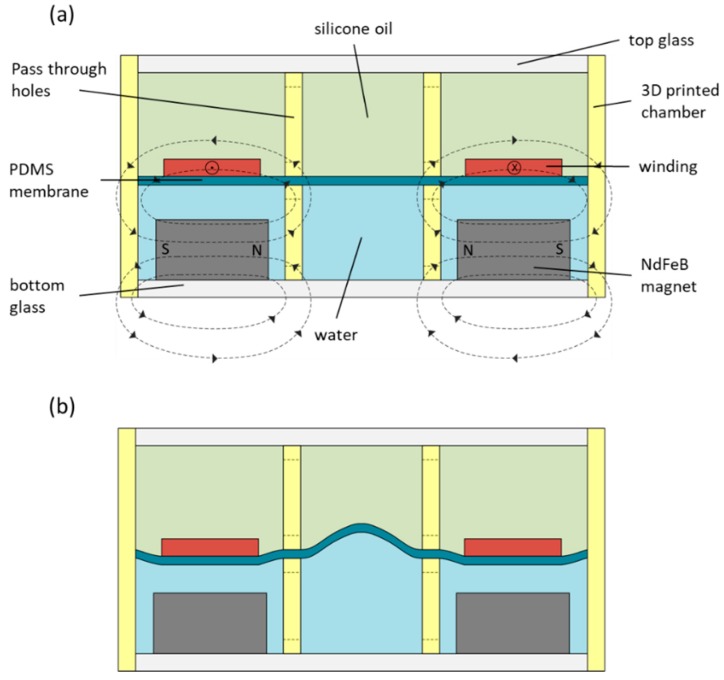
(**a**) Cross section of the magnetically actuated liquid tunable lens. Radial magnetic fields are shown as dashed lines and arrows in the magnet show the direction of the field. The cross and dot in the winding show current direction (into page and out of page, respectively). (**b**) The lens in the actuated state, showing a deformed membrane in the middle, which forms the liquid lens in the presence of electrical current.

We have designed a liquid lens comprised of two chambers of density matched liquids of different indices of refraction separated by a polydimethylsiloxane (PDMS) membrane, shown in Figure 1. Here, the two liquids are silicone oil (index of refraction *n_s_* = 1.49) and water (*n_w_* = 1.33). The lens itself is located at the central circular region of the two chambers. The area surrounding this central region holds a permanent magnet with a radial magnetic field on one side of the PDMS membrane, and a flat, circular winding of wire on the other side of the membrane. Compared to a previously reported magnetically actuated design [37] that is based on axial magnetic field, the proposed magnetic circuit is simpler. Further, the extent of the magnetic field through which the winding extends itself to its surroundings is smaller, which reduces any complications that might arise from possible mutual inductance with the surroundings. Finally, our design improves the coupling between the permanent magnet and the winding, resulting in a more efficient transducer.

To define the aperture of our lens, the membrane is pinned in place by a ring of posts that hold the membrane in place at the edges of the lens, but allow liquid to pass back and forth between the central region where the lens is located and the outer regions, where the winding and permanent magnet are housed.

When current is run through the winding, the movement of charge through the magnetic field of the permanent magnet generates a force, known as the Lorentz force. The Lorentz force for a point charge is given by Equation (1)

(1) $$\vec{F}=q\vec{v}\times \vec{B}$$

where *q* is the value of the charge, *v* is the velocity of the charge, and *B* is the magnetic field through which it moves. In the case of our system we can generalize this Lorentz force in the following equation (Equation (2))

(2) $$\vec{F}=\oint I\;d\vec{l}\times \vec{B}$$

where the integral is calculated throughout the length of the wire, *I* is the current, *dl* is the differential length of the winding cross section (which defines the direction the current is constrained to flow in), and *B* is again the magnetic field.

We can induce a steady current through the winding by applying a direct current (DC) voltage across the length of wire that composes the winding. As demonstrated in Figure 1, when a current is run counterclockwise (when viewed from the top) through the winding, the Lorentz force generated will attract the winding down towards the magnet, which is fixed in its housing. Since the PDMS membrane is between the winding and the magnet (and the winding is affixed to the membrane), the movement of the winding in response to the exerted Lorentz force displaces water that previously filled the region between the winding and the magnet. Since liquids are incompressible, this displaced volume of water is simply transferred to the central region of the chamber, where the membrane bulges upwards by a volume equal to the volume displaced by the attractive force to form the lens, as seen in Figure 1b. Since the index of refraction of silicone oil is greater than that of water (1.49 and 1.33, respectively), a diverging lens is formed.

If the direction of the current is reversed (i.e., if it is applied to run in a clockwise direction), then the cross product means that the direction of the Lorentz force is reversed as well, and the winding is repelled by the permanent magnet instead of attracted to it. This displaces the silicone oil in the region of the chamber above the winding into the central section of the chamber, causing the membrane to bulge downwards there. This results in a converging lens. Thus, the power of the lens is correlated to the amount of current running through the winding, where higher current leads to a higher-power lens, and the sign of the lens (whether it is diverging or converging) is determined by the direction of the current.

Other factors that will affect the power of the lens at a given current are the strength of the magnetic field, which depends on the material/strength of the permanent magnet, and the distance of the winding from that magnet. The thickness of the PDMS membrane will also affect the power of the lens, since a thicker membrane will be stiffer, and thus require a stronger force to bend. Finally, the amount of current that we can run through our winding will be limited by ohmic heating. In our design, we have tried to carefully balance these different factors.

## 3. Fabrication

**Figure 2:**
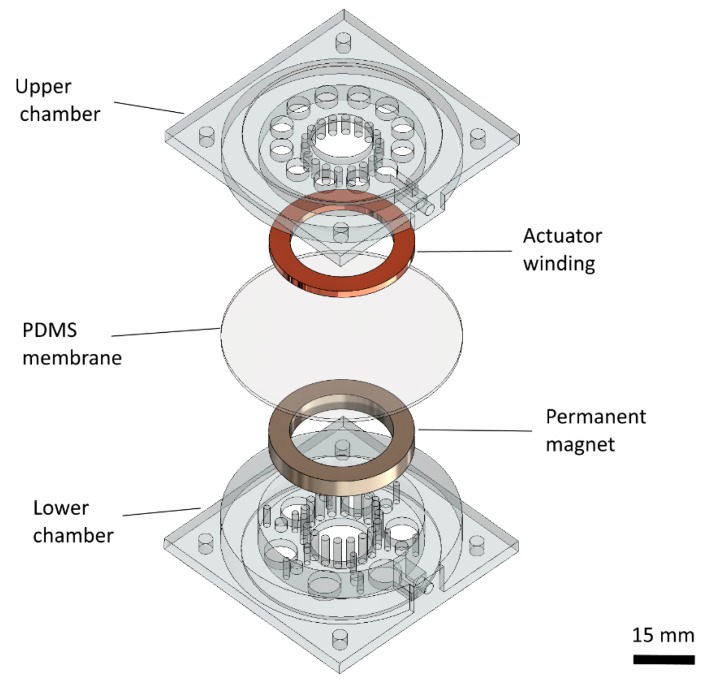
Exploded view of the magnetically actuated liquid tunable lens, showing 3D printed upper and lower chamber, permanent ring magnet, copper wire winding, and polydimethylsiloxane (PDMS) membrane to define separate chambers for water and silicone oil.

In order to fabricate the lens, we chose to make the lens housing via 3D printing. Specifically, we used a stereolithography (SLA) 3D printer that has the ability to print photopolymer resin with an *XY* resolution of 150 μm and a layer thickness of 100 μm (Form 2, Formlabs Inc., Somerville, MA, USA). The lens housing consists of two separate 3D prints, the upper chamber, and the lower chamber (see Figure 2). The primary difference between the upper and lower chamber is that the lower chamber has a recessed holder where the permanent magnet can be attached and is thicker (to accommodate the thickness of our magnet). Since even the “clear” photopolymer resins used in SLA are not optically transparent, we left the top and bottom facets of the 3D printed chambers open so that two-inch sapphire wafers can be glued in place instead to form the upper and lower facets of our liquid chambers.

The winding used in our design was made by taking a 30 American wire gauge (AWG) copper wire with nylon insulation and winding it into a ring with a power drill and a custom jig. The jig helped ensure that the winding ring was tightly packed, at which point a silicone-based adhesive diluted with hexane was poured in. The hexane evaporated and the silicone cured, resulting in a ring of copper winding with an inner diameter of 25 mm, an outer diameter of 35 mm, and a thickness of 2 mm. The winding used 10 m of wire and had approximately 125 turns. After the adhesive set, the winding was removed from the jig.

**Figure 3:**
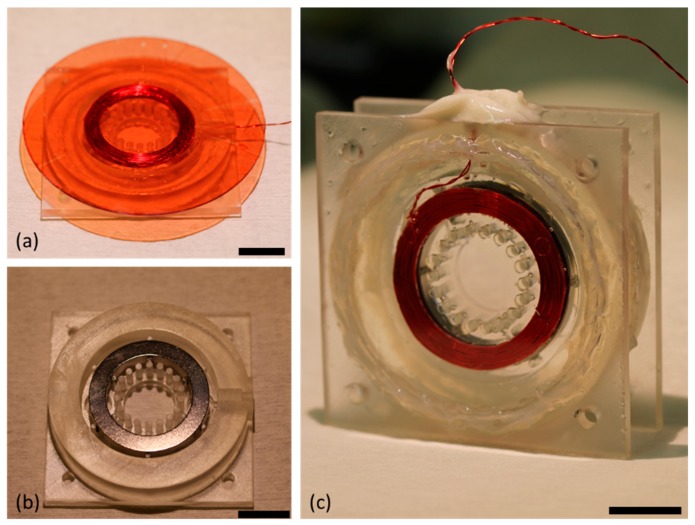
Photographs of magnetically actuated lens at different stages of fabrication. Scale bars represent 15 mm. (**a**) The upper half of the 3D printed housing showing the PDMS membrane on a glass dummy wafer on top, which has been glued to the winding and the housing, seen on bottom. (**b**) The lower half of the 3D printed housing, showing the magnet mounted in place. (**c**) Fully assembled lens showing two halves of the housing glued together and a 2-inch sapphire wafer sealed against both optical facets of the lens (sealed ports on the top can be seen as the white glued area). The diameter of the liquid lens is defined by the ring of posts inside the winding.

At this stage we were ready to begin the assembly of the lens. This was done in two stages, first by assembling the top portion consisting of the upper chamber, actuator winding, and PDMS membrane, followed by assembling the bottom portion consisting of the permanent magnet and lower housing. Once they were both completed, they could be combined and filled with the two liquids to form the final lens. Fabrication of the top portion of the device required that we first create a thin PDMS membrane (approximately 40 μm thick) on a 3-inch dummy wafer made of glass. This was done by first spin coating a sacrificial layer of photoresist (AZ P4620, MicroChem Corp., Westborough, MA, USA) onto the wafer and soft baking it. After the soft bake was complete, PDMS was spin coated on the wafer at 3000 rpm for 30 s. The wafer was then transferred to a hot plate set at 100 °C while the PDMS cured. Once the PDMS membrane had fully cured, the actuator winding was glued to the central portion of the membrane using a silicone adhesive (Sil-Poxy, Smooth-On, Macungie, PA, USA). The PDMS and winding on the dummy wafer were then fit into the upper chamber of the housing, and the outer rim that defined the upper liquid chamber was also affixed to the PDMS membrane using Sil-Poxy. This step can be seen in Figure 3a.

While the silicone adhesive was allowed to cure, the permanent magnet was also fit into the lower housing and glued into place so that it would remain stationary when the lens is in operation (see Figure 3b). The magnet used was a neodymium magnet (NdFeB, type 45 with maximum flux density of 1.33 T) with an inner diameter of 25 mm and an outer diameter of 35 mm, which was magnetized such that north was on its inner radius and south was on the outer radius. The final step in preparing the two halves of the lens was to remove the PDMS membrane from the dummy wafer. This was done by submerging the upper half of the lens in a beaker of acetone with gentle agitation until the wafer lifted clear of the membrane. At this stage the two halves were glued together using epoxy, and once that epoxy had set epoxy was again used to attach the 2-inch sapphire wafer to the outside of both halves of the chamber to act as the facets of the liquid lens.

At this point the lens was ready to be filled. This was done by slowly injecting water and oil into the two chambers through the ports built into the 3D printed housing. We filled the bottom chamber of the lens with water and the top chamber with silicone oil, and then sealed the chamber entirely using a marine-grade epoxy, designed to cure in the presence of liquid. The completed liquid lens is pictured in Figure 3c.

## 4. Results

In order to characterize our magnetically actuated liquid lens, we first measured the focal length versus voltage. We chose to report applied voltages instead of applied current because we used a DC voltage source for our experiments (Agilent E3644A); however, the current follows the voltage very linearly by the well-known relation *I* = *V*/*R*, where the resistance of our system (the resistance of the copper winding) can be considered a constant for our purposes. In order to make measurements of the focal length of the lens, we needed a single setup that could measure the focal length of both a diverging lens (formed when we applied a negative DC bias that results in a clockwise current) and of a converging lens (formed when we applied a positive DC bias, resulting in a counter-clockwise current).

**Figure 4:**
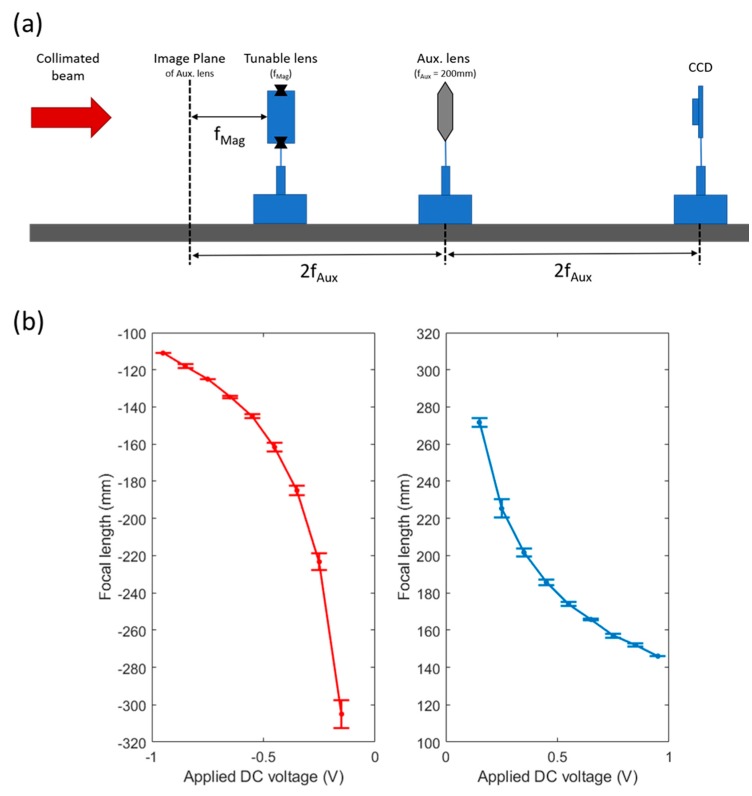
(**a**) A diagram of the experimental setup for measuring the focal length of the tunable lens. A collimated laser beam acts as an object at infinity and an auxiliary lens with a 200 mm focal length is placed at a distance of twice of that focal length from a charge coupled device (CCD) detector and both are fixed in place. Then the tunable lens is allowed to slide along an optical rail and is adjusted until the light focuses to a point. The distance between the lens and the virtual object is the focal length. (**b**) The graph of lens focal length vs. applied voltage, with error bars showing standard deviation of the measurement under cycling. When negative voltages are applied the winding is attracted to the magnet and a concave lens (corresponding to a negative focal length) is formed. When positive voltages are applied, the direction of current flow is reversed, meaning the winding is repelled by the magnet and a convex (positive focal length) lens is formed.

A diagram of the optical setup we chose to implement for this purpose is shown in Figure 4a. It consists of four components on a single, long optical rail: a 1.3 megapixel charge coupled device (CCD) sensor (CGN-C013-U, Mightex Systems, Toronto, ON, Canada), an auxiliary lens—in this case a plano-convex lens with a focal length equal to 200 mm, the tunable lens itself, and a collimated beam from a HeNe laser. All components were aligned on the optical axis defined by the laser beam, and then the auxiliary lens was placed at a distance of twice its focal length from the CCD. On the other side of the auxiliary lens, a resolution target was temporarily inserted at twice the focal length, at which point fine adjustments were made to the system such that the image of the target was brought into best focus on the CCD. The location of the target was marked on the optical rail as the image plane, and then the target was removed from the system and the tunable lens was inserted.

Voltage was applied to the winding of the tunable lens ranging from −0.95 V to 0.95 V at intervals of 0.1 V. For each voltage, the position of the tunable lens was adjusted until the laser beam was again focused to the smallest possible point on the CCD. The distance between the marked image plane and the facet of the tunable lens furthest from the CCD gave the focal length of the magnetically actuated lens and was recorded. When the lens was operating in the converging regime it would need to be positioned to the left of the marked image plane, so that the collimated beam would pass through it, focus to a point at the image plane of the auxiliary lens, and then be relayed to the CCD. This yielded a positive focal length measurement. When the lens was operating in the diverging regime it would need to be positioned to the right of the marked image plane, such that the virtual point image formed by the liquid lens would be located at the marked image place. This yielded a negative focal length.

Graphs of measured focal length vs. applied voltage can be seen in Figure 4b. The measurement of the focal length for each applied voltage was repeated over 3 cycles in order to get an idea of the error of the measurement. It is worth noting that we did not see any hysteresis during voltage increase or decrease cycles. The error bars of the graphs report the standard deviation of each measurement set. As we would expect, the error is greater at smaller applied voltages because such small amounts of actuation led to very large radius of curvature of the lens, as shown by the Lensmaker’s Equation (3)

(3) $$\frac{1}{f}=\frac{n_{1}-n_{2}}{R}$$

where *f* is the focal length, *R* is the radius of curvature, and *n’s* are the indices of refraction. At very large radii, the focal length of the lens becomes highly sensitive to even small changes of applied force. We can also see in Figure 4b that the focal length range is greater when the lens is operating in the divergent regime as compared to when operating in the convergent regime. This occurs because in the divergent case the Lorentz force attracts the winding closer to the magnet where the magnetic field is strong, while in the convergent case the Lorentz force repels the winding away from the magnet, taking it to a region of weaker magnetic field.

**Figure 5:**
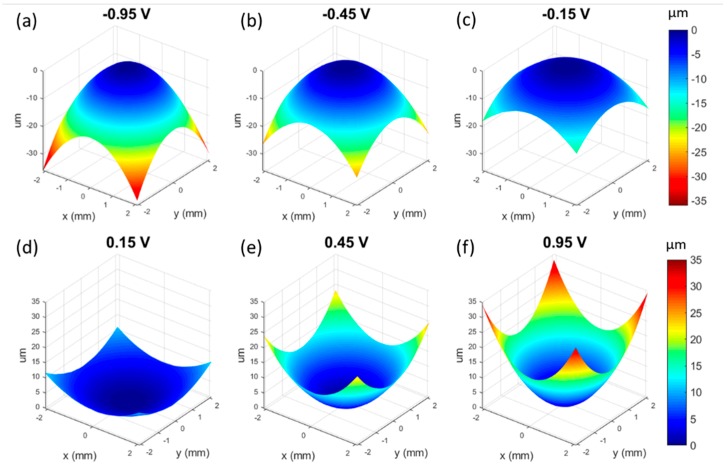
Shack–Hartmann wavefront sensor data from the tunable lens at different levels of actuation. (**a**–**c**) show the lens concave (diverging), where the increasingly large negative applied voltages lead to a concave lens with an increasing optical power. (**d**–**f**) show the lens convex (converging) where increasing applied voltages lead to an increasing optical power of the lens.

The next measurement we conducted was to measure the shape of the wavefront produced by our tunable lens (Figure 5). This was done using a Shack–Hartmann wavefront sensor (SHWS) equipped with a HeNe laser with a wavelength of 632 nm. Our SHWS (WFS150C, Thorlabs Inc., Newton, NJ, USA) setup allowed us to measure the shape of the central 4.75 mm region of our 15 mm diameter lens. At zero volts applied and no current through the wire, we would expect the wavefront to be essentially flat. As we began to apply voltage, we observed an increasing curvature to the wavefront shape. The *z*-axis of the wavefront sensor pointed towards the sensor, so that when the impinging wavefront was diverging (concave lens) the shape of the plotted wavefront was mounded, with peak values in the center region. When we changed the direction of the current, the wavefront became converging (convex lens) and the shape of the plotted waveform was cupped, with peak values at the outer region. This data can also be fitted with Zernike polynomials in order to describe the aberrations of the lens; the results are shown in Table 1. The defocus/power term in the table is related to the curvature of the wavefront, which is proportional to the curvature of the lens itself, and we see the expected trend from large negative number to large positive number as we move from large negative applied voltages to large positive applied voltages. The Zernike data confirms that aberrations are generally small, and the most significant of them seems to be astigmatism. This may be a result of asymmetries caused by possible slight warping of the 3D printed housing where it pins the edge of the PDMS membrane, or inconsistency of the membrane thickness.

**Figure 6:**
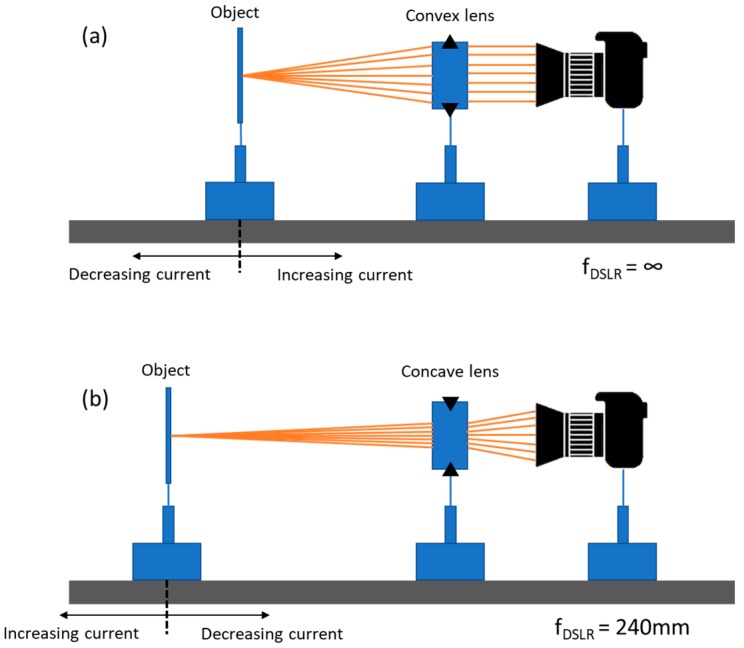
(**a**) A diagram of the optical setup used to image a resolution chart and a mascot logo image when the tunable lens is operating in the converging regime (convex lens). A digital single-lens reflex (DSLR) camera with its focal length set to infinity is placed on one side of the tunable lens, and an optical object is moved until it comes into the clearest focus. (**b**) A diagram of the optical setup used when the tunable lens is operating in the diverging regime (concave lens). The focus of the DSLR is set to 240 mm, and the optical object is moved until it comes to the clearest focus. Since a stronger (more diverging) lens will shift the overall focus of the system to be longer, the object must be moved further away as the current through the tunable lens increases.

**Figure 7:**
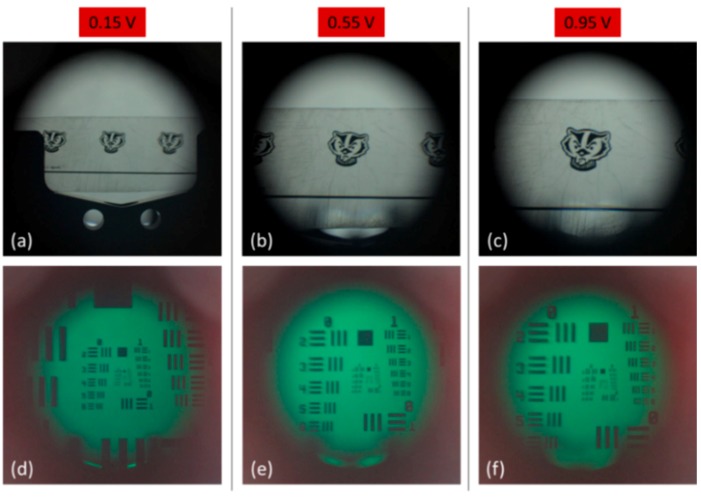
Images from the tunable lens operating in the convex regime. (**a**–**c**) show the image of a University of Wisconsin mascot logo, magnified with increasing applied voltage, and (**d**–**f**) show the same trend but with a 1951 US Air Force resolution target. The resolution was observed to increase along with the applied voltage (which corresponds to the focal length and thus f/# of the lens), allowing us to resolve line pairs corresponding to 3.56 lp/mm at 0.15 V, 4.49 lp/mm at 0.55 V, and 7.13 lp/mm at 0.95 V.

The final step in characterizing the optical properties of our magnetically actuated lens is to use the lens for imaging and observe its performance. Due to the inherent differences of converging and diverging lenses, two different setups were required to capture the performance of our tunable lens in these two separate regimes, though both utilized a Canon digital single-lens reflex (DSLR) camera fitted with a macro lens (see Figure 6). To observe the image created by the lens in the converging regime, we mounted the DSLR on an optical rail and manually set its focus to infinity. Our tunable lens was then placed a short distance in front of the DSLR, and a 1951 US Air Force resolution target was placed at the focal length of the tunable lens. For each change in the applied voltage, the target was moved to the new focal length of the tunable lens and a series of images were captured. This was done both with an image of a University of Wisconsin mascot logo as well as with a 1951 US Air Force resolution target, the results of which are shown in Figure 7. We observed the magnification of the image with increasing applied voltage and also measured the resolution of the system at each voltage. The maximum resolution reached was 7.13 line pairs per mm (lp/mm) and the resolution increased as the f/# decreased, as we would expect.

**Figure 8:**
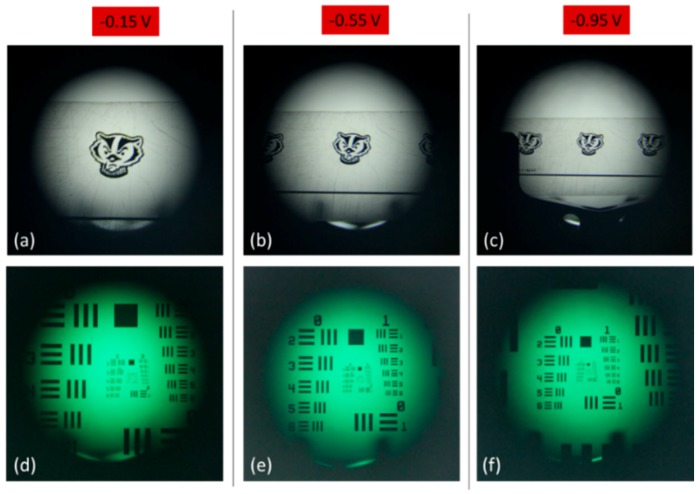
Images from the tunable lens operating in the concave regime. (**a**–**c**) show the image of a mascot logo, demagnifying further with the increase of the absolute value of negative applied voltage, and (**d**–**f**) show the same trend but with a 1951 US Air Force resolution target.

Our imaging setup was then slightly modified to work in the diverging regime of our tunable lens. The manual focus of the DSLR was set to a distance of 240 mm, which placed it on the far side of our tunable lens. Then, when a negative voltage was applied to the lens so that it began to diverge, the tunable lens effectively modulated the focal length of the overall system, such that the more voltage applied, the farther away the focal point of the system would be shifted (see Figure 6b). For each voltage applied to the lens, an optical target was moved until the best focus was reached. This was done both with an image of a mascot logo and with a 1951 US Air Force resolution target. The resulting images can be seen in Figure 8. As we would expect for an increasingly powerful concave lens, the image of the optical targets shrink as more current is applied to the tunable lens system.

## 5. Conclusions

A magnetically actuated liquid lens was developed to function as a tunable-focus lens that operated in both converging and diverging regimes. A winding of copper magnet wire and an NdFeB magnet created a Lorentz force when a current flowed through the winding, and the resulting displacement of liquid corresponded to a change in the focal length of our lens, either concave or convex depending on the direction of current. We characterized the range of focal lengths achievable by the lens, observing smooth, asymptotic curves for both regimes of operation.

We also measured and characterized the wavefront produced by the lens as it was actuated using a Shack–Hartmann wavefront sensor. The resulting data was fit with Zernike polynomials, the coefficients of which were also reported. We observed very low spherical aberration, and the most significant contributor to aberrations was observed to be astigmatism, which may be from defects in the lens housing, or defects introduced during the fabrication process. Overall, aberrations were small, indicating a good lens quality. Finally, we demonstrated the imaging capability of the lens in both concave and convex regimes, utilizing a DSLR camera with the lens, observed the focal length tuning, and measured the resolution of the lens, which was found to be as high as 7.13 lp/mm.

After demonstrating the proof of concept, we now aim to reduce the size of the device to make it more practical for miniaturized imaging systems. Further, relatively thick PDMS used in this device, although mechanically robust, will require more pressure to deform compared to thinner membrane. Thus in future downsizing, we will use thinner PDMS membrane. From the standpoint of actuators, we plan to use smaller permanent magnets. Further, we will employ a smaller winding with a higher number of turns to provide enough wire length needed to generate adequate force. With these alterations, we believe that it is practical to downsize the current device to sub-20-mm diameter for overall packaging.

## Figures and Tables

**Table 1:** Zernike coefficients (in μm) of our lens at different applied voltages, corresponding to the wavefronts shown in Figure 5.

Index	Mode Name	Zernike Coefficients					
		−0.95 V	−0.55 V	−0.15 V	0.15 V	0.55 V	0.95 V
1	Piston	5.680	4.816	2.350	−2.047	−3.979	−5.291
2	Tilt y	0.676	0.511	0.444	−0.325	−0.945	−0.813
3	Tilt x	0.180	−0.004	0.454	−0.005	−0.349	−0.513
4	Astigmatism ± 45°	−0.140	−0.123	−0.106	0.122	0.221	0.251
5	Defocus/Power	−6.380	−5.226	−3.259	2.062	4.804	6.137
6	Astigmatism 0/90°	−0.047	0.007	0.074	0.146	0.183	0.186
7	Trefoil y	−0.001	−0.009	0.008	0.023	0.003	−0.014
8	Coma x	−0.015	−0.012	−0.010	0.006	0.020	0.025
9	Coma y	0.007	0.010	0.012	−0.020	−0.019	−0.025
10	Trefoil x	−0.005	0.003	0.012	0.018	0.039	0.042
11	Tetrafoil y	0.017	0.013	0.006	0.011	0.003	0.000
12	2nd astigmatism y	0.009	0.005	0.005	0.001	−0.005	−0.003
13	Primary spherical	0.000	−0.001	−0.001	0.001	0.019	0.026
14	2nd astigmatism x	0.004	0.005	0.001	0.006	−0.007	−0.012
15	Tetrafoil x	0.014	0.004	0.010	0.004	0.011	0.004
